# Predicting depression severity using machine learning models: Insights from mitochondrial peptides and clinical factors

**DOI:** 10.1371/journal.pone.0320955

**Published:** 2025-05-14

**Authors:** Toheeb Salahudeen, Maher Maalouf, Ibrahim (Abe) M. Elfadel, Herbert F. Jelinek

**Affiliations:** 1 Department of Management Science and Engineering, Khalifa University, Abu Dhabi, UAE; 2 Department of Computer and Communication Engineering, Khalifa University, Abu Dhabi, UAE; 3 Center for Secure Cyber Physical Systems, Khalifa University, Abu Dhabi, UAE; 4 Department of Medical Sciences and Health Engineering Innovation Group, Khalifa University, Abu Dhabi, UAE; 5 Biotechnology Center, Khalifa University, Abu Dhabi, UAE; Sarich Neuroscience Research Institute, AUSTRALIA

## Abstract

Depression presents a significant challenge to global mental health, often intertwined with factors including oxidative stress. Although the precise relationship with mitochondrial pathways remains elusive, recent advances in machine learning present an avenue for further investigation. This study employed advanced machine learning techniques to classify major depressive disorders based on clinical indicators and mitochondrial oxidative stress markers. Six machine learning algorithms, including Random Forest, were applied and their performance was investigated in balanced and unbalanced data sets with respect to binary and multiclass classification scenarios. Results indicate promising accuracy and precision, particularly with Random Forest on balanced data. RF achieved an average accuracy of 92.7% and an F1 score of 83.95% for binary classification, 90.36% and 90.1%, respectively, for the classification of three classes of severity of depression and 89.76% and 88.26%, respectively, for the classification of five classes. Including only oxidative stress markers resulted in accuracy and an F1 score of 79.52% and 80.56%, respectively. Notably, including mitochondrial peptides alongside clinical factors significantly enhances predictive capability, shedding light on the interplay between depression severity and mitochondrial oxidative stress pathways. These findings underscore the potential for machine learning models to aid clinical assessment, particularly in individuals with comorbid conditions such as hypertension, diabetes mellitus, and cardiovascular disease.

## 1 Introduction

Recent research indicates an increase in neuropsychiatric disorders [1]. This makes it crucial to better understand these conditions and their complex interactions with chronic diseases, as well as associated biochemical markers for improved personalized treatment and outcomes [2]. Depression is the most common chronic stress-related disorder, affecting 20% of people at some point in their lives [3]. According to the World Health Organization (WHO), this disorder is responsible for approximately 800,000 deaths annually and is one of the leading causes of suicide [4].

Several studies have indicated a possible role of mitochondrial oxidative stress in the development of depression severity—the intensity and number of depressive symptoms and functional impairments [5]—and other mental health problems. The severity of depression is commonly assessed using standardized tools such as the Hamilton Depression Rating Scale (HAM-D) or the Patient Health Questionnaire (PHQ-9) [5]. Understanding and measuring the severity of depression is crucial to determine the appropriate treatment approach, [6] and assessing the severity of depression [7]. Incorporating mitochondrial peptides in the assessment of the severity of depression holds significant promise [8]. Biomarkers can provide objective measures of depression, assist in identifying disease in a timely manner, predict treatment responses, and identify subtypes of depression [3]. Furthermore, mitochondrial biomarkers may provide information on the underlying biological mechanisms of depression, which can lead to more personalized and effective treatment strategies [9]. Integrating biomarkers in the evaluation of the severity of depression increases the number of factors taken into account for diagnosis and avoids possible misdiagnosis or under-/over-prescribing [10].

However, information on the role of specific mitochondrial-associated peptides is still lacking [11–19]. This study aims to fill this critical gap by: (1) examining the impact of Mitochondria-derived peptides (MDPs), such as Humanin, MOTS-c, and p66Shc, together with ACE genotypes in the context of cardiovascular disease (CVD), hypertension (HT), and diabetes mellitus (DM); (2) clarifying the intricate interplay between these mitochondrial factors and the progression of depressive disorders, and advance understanding of the role of mitochondrial-derived oxidative stress peptides in the progression of depressive disorders.

### 1.1. Mitochondrial oxidative stress peptides

**Fig 1:**
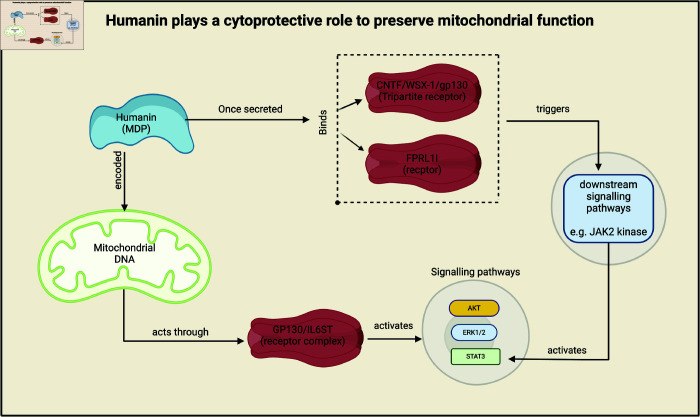
Pathways of humanin.

Mitochondria-derived peptides (MDPs), include humanin (HN) and mitochondrial ORF (Open Reading Frame) of 12S rRNA Type C (MOTS-c), are encoded by mitochondrial DNA, and play crucial roles in maintaining mitochondrial function [20,21]. MDPs have been associated with neuroprotection and cardioprotection, affecting the development of cardiovascular disease [13]. Humanin (HN) has also been shown to be involved in major depressive disorder [22]. HN affects mitochondrial oxidative phosphorylation, adenosine triphosphate (ATP) production, and inhibition of apoptosis, by influencing glucose metabolism [23]. Fig 1 describes and displays the HN pathways. HN binds to two types of receptors and activates signaling pathways: protein kinase B (AKT), extracellular signal-regulated kinase 1/2 (ERK1/2) and signal transducer and transcription activator 3 (STAT3)—a transcription factor that plays a critical role in mediating cellular responses to cytokines and growth factors. It is involved in various biological processes, including cell growth, differentiation, and inflammation, which in turn trigger several downstream signaling pathways, such as Janus kinase 2 activation (JAK2), a non-receptor tyrosine kinase involved in signaling by cytokines, which subsequently activates STAT3 [24,25].

**Fig 2:**
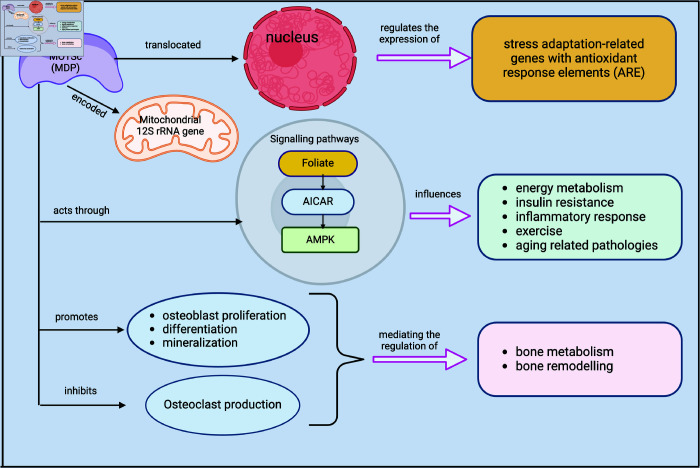
Pathways of MOTS-c.

MOTS-c also has various physiological functions (as shown in Fig 2 such as reducing insulin resistance, preventing obesity, and regulating bone remodeling, primarily through the Folate-AICAR-AMPK pathway. The Folate-AICAR-AMPK pathway is a series of interconnected chemical reactions in the body that involve the molecules folate, AICAR (5-aminoimidazole-4-carboxamide ribonucleotide), and AMPK (Adenosine Monophosphate-activated protein kinase). Folate is a type of B vitamin that is important for various cellular functions, including the production of DNA and other genetic material. AICAR is a compound that can activate the AMPK protein, which is involved in the regulation of energy levels in cells. When AICAR activates AMPK, it can influence various processes in the body, such as cell growth, metabolism, and responses to stress [26]. This pathway is important for understanding how the body regulates energy and other essential functions at the cellular level. The expression and function of MOTS-c have been implicated in the pathogenesis of various diseases [23,27,28]. Similarly, Mots-c has been associated with psychiatric disease, but further research is required to determine any connection to major depressive disorder [29].

**Fig 3:**
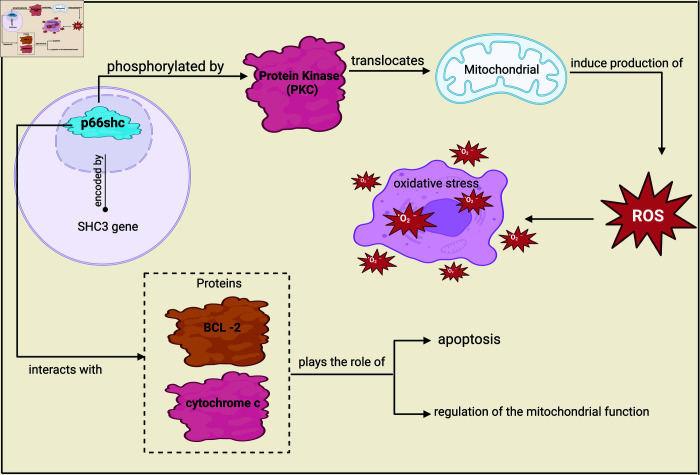
Pathways of p66shc.

p66shc is a nucleus-derived peptide but acts on the mitochondrial electron transport chain [30,31]. It serves as a redox enzyme that regulates reactive oxygen species (ROS)^1^ metabolism and apoptosis, influencing cell injury pathways [32–34]. Its role in cardiovascular pathophysiology is well established [35,36]. Fig 3 shows the pathways of p66Shc, where Protein Kinase C (PKC), a family of enzymes that play a crucial role in cellular signaling pathways, phosphorylates p66shc and translocates to mitochondria, thus promoting ROS production. Dysregulation of p66shc expression and function has been implicated in the pathogenesis of various diseases and psychopathology [30,37–39].

### 1.2. Angiotensin-converting enzyme (ACE) genotype and depression

The ACE genotype has been shown to be associated with depression in several studies, including variants of the ACE gene linked to cortisol secretion and late life depression [40,41]. Furthermore, the ACE genotype has been associated with cardiovascular disease, diabetes [42], cardiac autonomic neuropathy as part of diabetes progression [43], COVID-19, and recurrent aphthous stomatitis [40,41]. In addition, mice lacking ACE have been shown to have a lower systolic blood pressure [44]. In particular, the ACE DD genotype is associated with increased plasma ACE concentrations, which affects the conversion of angiotensin I to angiotensin II [41,45]. However, the precise nature of the relationship between the ACE genotype and depression remains a subject of ongoing debate, necessitating further investigation [41,46].

### 1.3. Clinical factors in depression

Research has confirmed the coexistence of depression and cardiovascular disease (CVD). Patients recovering from a myocardial infarction often experience depressive symptoms, which complicates the prognosis of CVD, including heart failure and [47–50]. This association is influenced by shared risk factors, unhealthy lifestyles, and chronic diseases [49]. Mental stress can negatively affect heart health, increasing the risk of adverse cardiac events [51,52]. Depression in patients with heart failure leads to higher hospital readmission and mortality risks due to its association with heart attacks and blood clots [53]. Hypertension and diabetes, common chronic diseases, are also linked to CVD and depression [48,54]. Despite the well-established connection between CVD and depression, the underlying mechanism remains largely unknown [55,56]. The complex relationship between depression, CVD, hypertension, and diabetes requires further research for effective management strategies.

The potential roles played by Humanin, MOTS-c, p66shc, and the ACE genotype have remained largely unexplored, representing a significant gap in understanding depression and disease progression. This study aims to fill this critical gap by examining the impact of Humanin, MOTS-c, p66Shc, and ACE genotypes in the context of cardiovascular disease (CVD), hypertension (HT) and diabetes mellitus (DM). This research sought to clarify the intricate interplay between these mitochondrial factors and the progression of depressive disorders, shed light on potential avenues for therapeutic interventions, and advance understanding of the role of mitochondrial-derived oxidative stress peptides in the progression of depressive disorders.

## 2. Methodology

### 2.1. Data description

The data set used in this study was obtained from a public screening program (DiabHealth) between 2002 and 2015, which was approved by the Human Research Ethics Committee (ethics approval number: 2006/042) and followed the Helsinki protocol. All participants gave their informed consent in writing to participate in this study. The data set consists of 261 screening reports with patient ages ranging from 25 to 92 years. To limit the data points reported from the available clinical report, the last reported entry was used for each patient when there were multiple attendances. Therefore, the final data set consisted of 830 individuals. Humanin, MOTS-c, p66Shc, ACE genotype, gender, age, medication use, CVD, hypertension (HT) and type 2 diabetes mellitus characteristics were extracted. Depression scores were determined using the Patient Health Questionnaire (PHQ-9). The PHQ-9 score of 5 categories or classes of depression is shown in Table 1. The higher the score, the more symptoms of depression there are and the more severe the depression.

**Table 1:** Depression severity level with corresponding PHQ-9 scores

Depression severity	PHQ-9 score
Normal or no depression	0–4
Mild depression	5–9
Moderate depression	10–14
Moderately severe depression	15–19
Severe depression	≥20

### 2.2. Data preprocessing

The data set consists of 13 variables, including the target variable (the PHQ-9 score). Predictor variables include four biomarkers (Humanin, MOTS-c, p66shc, and ACE levels), gender, birth age, use of other medications, including antidepressants, CVD, hypertension (HT), use of HT medication, diabetes mellitus (DM), and use of DM medication. Feature selection was carried out using the Pearson correlation test.

However, there are more advanced and robust system of feature selection that could be used. One of such is the Boruta selection technique. The Boruta feature selection technique is an all-relevant feature selection method designed to identify all the important features in a dataset. It works by creating shadow features, which are duplicates of the original features with randomly shuffled values. A random forest classifier is then trained on the dataset with both original and shadow features. The importance of each feature is measured, and features that have a higher importance score than the best-performing shadow feature are considered important. This process is iteratively repeated to ensure robust feature selection, effectively distinguishing between relevant and irrelevant features [57]. However, Boruta can sometimes eliminate features that might be relevant in a specific research context, making it less suitable for studies requiring the inclusion of specific types of variables. Specifically, in this study, upon using Boruta, only four features (Humanin, MOTSc, P66, and BirthAge) were reported as important. This limited selection of features does not align with the research focus, which aims to investigate the role of mitochondrial peptides in the presence of various clinical factors.

Additionally, the Boruta feature selection technique, while robust, may not be the best applicable technique in the context of this study. By its nature of comparing feature importance against randomized features, Boruta can sometimes lead to overfitting, especially if the dataset is not sufficiently large. Also, Boruta’s performance is highly dependent on the quality of the data. Noisy or irrelevant features can skew the results, leading to less reliable feature selection. In contrast, the Pearson correlation test offers some advantages for feature selection, in the context of this study. The Pearson correlation test is straightforward to implement and computationally less demanding compared to Boruta. It is effective at identifying linear relationships between features and the target variable. This can be particularly useful for initial feature selection in datasets where linear relationships are expected. Furthermore, by selecting features based on their linear correlation with the target variable, the risk of overfitting is reduced, making the model potentially more generalizable to new data.

Therefore, while Boruta might overly simplify the feature set by focusing on only a few variables, Pearson correlation allows for a more inclusive selection process. This inclusivity is crucial for the research as it focuses on the peptides within the broader context of clinical factors, ensuring that the complexity of depression severity is adequately captured and analyzed.

**Fig 4:**
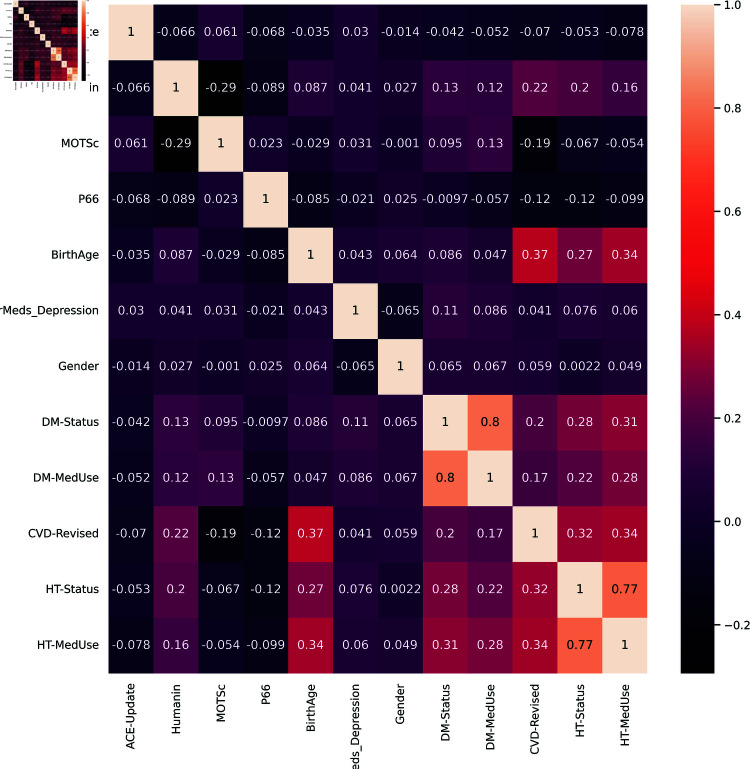
Correlation matrix of all twelve features.

**Fig 5:**
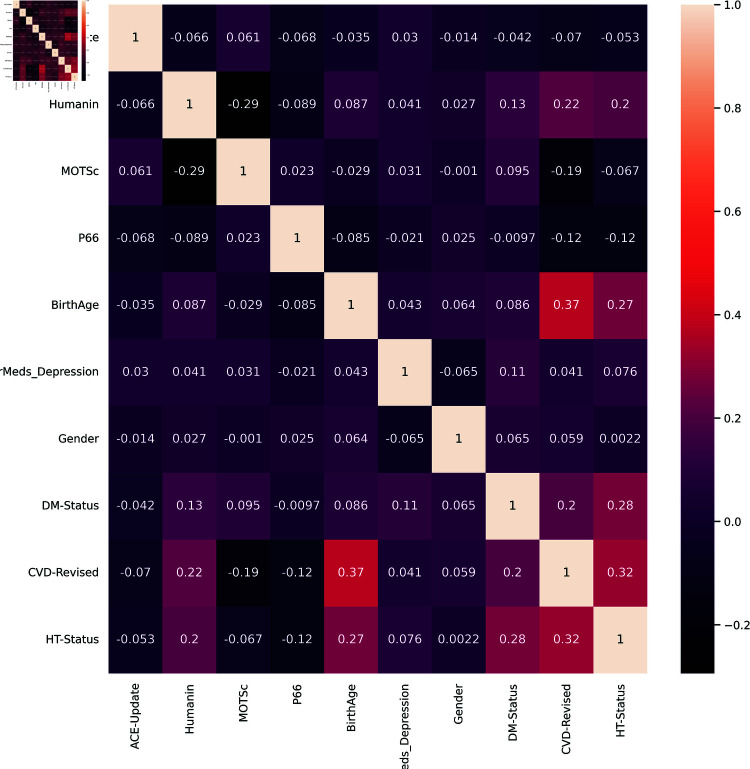
Correlation matrix of features after the dropping HT-MedUse and DM-MedUse.

Using the Pearson correlation test a threshold of 0.7 was used and, as evident in the correlation map in Fig 4, the DM status and HT status are highly correlated with DM-MedUse and HT-MedUse with values above the threshold of 0.7: 0.80 and 0.77, respectively. Consequently, DM-MedUse and HT-MedUse were removed to ensure that there is no high correlation between predictors, which reduced their number to ten as shown in Fig 5.

Depression classes were divided into binary classes (no depression and depression), three classes (no depression, mild depression, and severe depression), and five classes (from no depression to severe depression). The study adhered to the conventional multiclass division of five categories, as stipulated by the recognized standards of the Patient Health Questionnaire-9 (PHQ-9) score, which serves as a benchmark for assessing depression levels. However, the investigation conducted supplementary experiments to explore the feasibility of refining the classification scheme by reducing the severity levels of depression into binary and three-class divisions. Tables 2–4 show that there is an expected class imbalance for multiclass classification (5-class and 3-class) and binary classification.

**Table 2:** Class distribution for 5-class classification.

Depression severity	PHQ-9 score	Distribution
Normal or No depression	0–4	611
Mild depression	5–9	170
Moderate depression	10–14	29
Moderately severe depression	15–19	11
Severe depression	≥20	9

**Table 3:** Class distribution for 3-class classification.

Depression severity	PHQ-9 score	Distribution
Normal or no depression	0–4	611
Mild depression	5–14	199
Moderate depression	≥15	20

**Table 4:** Class distribution for binary classes.

Depression severity	PHQ-9 score	Distribution
Normal or no depression	0–4	611
With depression	≥20	219

Due to imbalanced data sets, a synthetic minority oversampling algorithm (SMOTE) [58] was included in the analysis when dealing with imbalanced data, with precision, recall, and F1 scores reported.

### 2.3. Machine learning algorithms

To classify the progression of depression, six different Machine Learning (ML) classifiers were used, including random forest (RF), support vector machine (SVM), logistic regression (LR), K-nearest neighbor (KNN), gradient boosting (GB), and artificial neural network, also known as multilayer perception (MLP).

After feature selection, grid search was used with k-fold cross-validation (k = 10) to adjust the hyperparameters of the classifiers. The data set was then divided into training and testing sets to avoid overfitting. This study used 80% of the sample data as training data and the remaining 20% as test data. The performance of the best-tuned models was validated using accuracy, precision, recall, and F1 score. Furthermore, the permutation importance was used to determine the importance of predictor variables by calculating their statistical contribution to the performance of the models.

**Fig 6:**
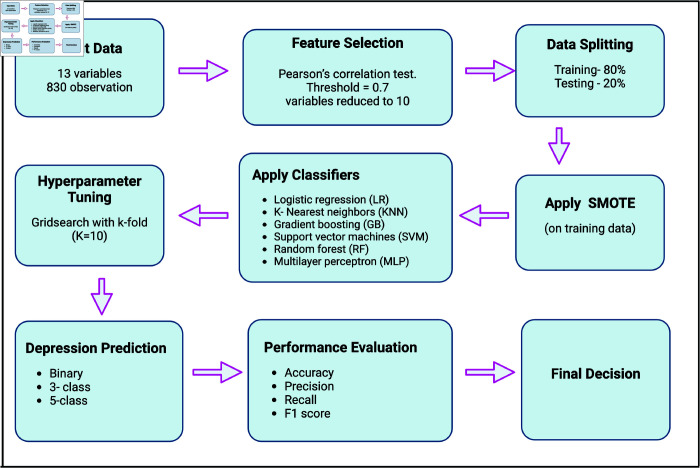
Flowchart for predicting depression severity.

All experiments in this study were performed with Python using the *scikit-learn* machine learning library version 1.4.0 and the *Tensorflow* 2.0 neural network library on a 16GiB RAM macOS Monterey machine with the Apple M1 Pro Chip. Fig 6 shows the flowchart of the steps used in this study to predict the severity of depression. Below is a brief description of the ML algorithms used.

#### 2.3.1. Random forest

Random forest (RF) classifiers fall under the broad umbrella of ensemble-based learning methods. They are simple to implement, easy to operate and have proven to be extremely successful in a variety of domains [59]. The key principle underlying the RF approach comprises the construction of many “simple” decision trees in the training stage and the majority vote (mode) across them in the classification stage. Among other benefits, this voting strategy has the effect of correcting for the undesirable tendency of decision trees to overfit training data. In the training stage, RF applies bagging to individual trees in the ensemble. Bagging repeatedly selects a random sample with replacement from the training set and fits trees to these samples. Each tree is grown without any pruning. The number of trees in the ensemble is a free parameter that is easily learned automatically using the so-called out-of-bag error.

RF is chosen for this study for its simplicity, ease of operation, and success across various domains. It is robust to overfitting due to the ensemble nature of combining multiple decision trees, and it performs well with imbalanced data as it can handle large datasets with higher dimensions and capture complex interactions between features.

#### 2.3.2. Support vector machine (SVM)

Support Vector Machine (SVM) is a powerful supervised learning algorithm that is used for classification, regression and outlier detection tasks. SVMs are particularly effective for solving binary classification problems, where the algorithm aims to find the best possible line or decision boundary (a hyperplane) that separates the data points of different classes [60]. This decision boundary is established in such a way that it maximizes the margin, which is the distance between the hyperplane and the closest data points in each category, making it easier to distinguish data classes. SVM can also be adapted for multiclass classification and regression tasks. SVM has different types and variants that provide specific functionalities, such as linear SVM and nonlinear SVMs. In this study, the RBF kernel SVM was used because it is capable of capturing complex relationships in the data, making it suitable for nonlinear classification tasks, especially in low-dimensional spaces.

Support Vector Machine (SVM) is selected for its effectiveness in binary classification problems and its versatility in handling linear and non-linear data through kernel functions, such as the RBF kernel. SVM is particularly effective for high-dimensional spaces, making it a robust choice for various classification tasks.

#### 2.3.3. Logistic regression

Logistic regression is a supervised learning classification algorithm that uses a logistic function to predict the probability of a target variable, typically binary in nature [61]. The algorithm works by computing the sum of the input features (including a bias term) and calculating the logistic of the result. The output of logistic regression is always between 0 and 1, which is suitable for binary classification tasks. The higher the value, the higher the probability that the current sample is classified as Class 1. Logistic regression can be adapted for multinomial classification by creating multiple binary classification problems. It is used in this study for both binary and multiclass classifications.

Logistic Regression (LR) is utilized for its suitability in predicting binary outcomes and its ability to adapt for multinomial classification. It is straightforward and interpretable, making it a good baseline model that works well with binary and multiclass classification problems.

#### 2.3.4. Gradient boosting

Gradient boosting (GB) is a form of machine learning boosting. It builds a sequential ensemble of predictors, each of which corrects the previous one. This strategy aims to adapt the new predictor to the residual errors of the old one. When the best future potential model is integrated with the past models, the total prediction error is reduced. The essential concept is to set the target results for the subsequent model to minimize the error. In gradient boosting, the target outcomes are dependent on the error gradient relative to the forecast. Each new model minimizes the prediction error for each training instance [62]. The goal for each case depends on how much the forecast changes the total error. If a small adjustment in the forecast reduces the error, the following target result will be high. The new model’s near predictions will decrease the error. However, if a small adjustment in the forecast does not affect the error, the target result is zero, and this forecast cannot be improved on[63].

Gradient Boosting (GB) is employed for its boosting technique, which builds a sequential ensemble of predictors that correct previous errors, thus minimizing prediction errors effectively. Known for its accuracy and ability to handle complex datasets, GB constructs models sequentially, correcting errors from previous models.

#### 2.3.5. K-nearest neighbor

The K-Nearest Neighbors (KNN) algorithm is a simple nonparametric supervised learning classifier that uses proximity to make classifications or predictions about the grouping of individual data points. It is typically used as a classification algorithm, working from the assumption that similar points can be found near each other [64]. It is easy to implement and understand, but has the major drawback of becoming significantly slower as the size of data grows. KNN works by finding the distances between a query and all the examples in the data, selecting the specified number of examples (K) closest to the query, and then voting for the most frequent label. Choosing the right K involves trying several K’s and picking the one that works best. In this study, the best K was 4.

K-Nearest Neighbors (KNN) is chosen for its simplicity and effectiveness in classification tasks, based on the proximity of data points. It works well with smaller datasets and is straightforward to implement. As a non-parametric method, KNN makes predictions based on the closest data points in the feature space, which can be beneficial for certain types of imbalanced data.

#### 2.3.6. Artificial neural network

Artificial Neural Network (ANN) is a machine learning algorithm inspired by the structure and function of the human brain. It is also known as a multilayer perceptron (MLP) connectionist model or parallel distributed processing system [65]. ANN is widely used for classification tasks such as image recognition, speech recognition, and natural language processing. ANN consists of multiple layers of interconnected nodes, or neurons, that process and transmit information. Each neuron receives input from other neurons, processes the information, and then passes the output to other neurons in the next layer. The output of the final layer represents the classification or prediction of the input data. ANN is known for its ability to learn from data and generalize to new data, making it a powerful tool for classification tasks. ANN has several types, including feedforward neural networks, convolutional neural networks, and recurrent neural networks, each with specific architectures and applications [66]. In this study, feedforward neural networks were used.

Artificial Neural Network (ANN) is selected for its powerful classification capabilities, particularly in tasks like image and speech recognition, due to its multilayered structure that mimics the human brain. ANNs can model complex patterns and interactions in the data, making them highly flexible and adaptable for various tasks.

The choice of the ML methods used in this study was guided by their proven effectiveness and suitability for the classification tasks at hand. They were chosen from different categories of algorithms, including linear (LR), nonlinear (SVM, KNN), ensemble (RF, GB), and neural network (ANN). This diversity in the application of machine learning methods helps in the prediction process by leveraging the strengths of various types of algorithms. Linear models like LR are simple and interpretable, making them good baseline models. Nonlinear models such as SVM and KNN handle complex relationships and interactions in the data. Ensemble methods like RF and GB improve prediction accuracy by combining multiple models. ANNs are powerful in modeling complex patterns due to their multilayered structure. This level of diversity ensures a comprehensive approach to predicting depression severity, accommodating the complexities and nuances within the dataset.

Table 5 outlines the criteria and reasons for selecting these machine learning methods, emphasizing their strengths.

**Table 5:** ML algorithms used in this study.

Type of Algorithm	Algorithm/method	Strengths/ reasons for selection
Linear	Logistic regression (LR)	• good baseline model that works well with binary
		• ability to adapt for multinomial classification
		• straightforward
		• Interpretable
Nonlinear	K-nearest neighbor (KNN)	• Simplicity and effectiveness in classification tasks, based on the proximity of data points
		• It works well with smaller datasets Straightforward to implement
	Support vector machine (SVM)	• Effectiveness in binary classification problems
		• Particularly effective for high-dimensional spaces
		• Versatility in handling linear and non-linear data through kernel functions
Ensemble	Randomforest (RF)	• Simplicity
		• Ease of operation
		• It is robust to overfitting due to the ensemble nature
		• It captures complex interactions between features It performs well with imbalanced data
	Gradientboosting (GB)	• Known for its accuracy and ability to handle complex datasets
		• It constructs models sequentially, correcting errors from previous models.
Neural Network	Artificial neural network (ANN)	• Ability to model complex patterns and interactions in the data
		• Highly flexible and adaptable for various tasks

## 3. Results

This study presented comprehensive results that shed light on the performance of the ML models. Specifically, detailed tabulated data for each class were provided in three distinct models, namely Model 1, Model 2, and Model 3. Model 1 consisted of all predictors and Model 2 consisted of biomarkers of oxidative stress (p66shc, Humanin and MOTS-c), ACE, age, and gender. Model 3 consisted only of biomarkers of oxidative stress. Model 3 was included to investigate the impact of oxidative stress biomarkers on the classification of severity of depression. To ensure accessibility to the performance of the three models, all result tables have been included in the supplementary section of this study. S9–S11 Tables in S1 Text show the results for all classes for Model 1, S12–S14 Tables in S1 Text for Model 2, and S15–S17 Tables in S1 Text for Model 3. Each table shows the result values for the six classifiers that were used in this study. The most pertinent results are summarized in Section [sec_3.1]3.2.

### 3.1. Understanding the data using explainable AI

In recent years, the application of explainable AI (XAI) techniques has become increasingly vital for interpreting complex machine learning models, particularly in high-stakes domains such as healthcare, finance, and social sciences [67]. This study leverages several XAI methods, including permutation importance [68], Local Interpretable Model-agnostic Explanations (LIME) [69] and SHapley Additive exPlanations (SHAP) [70], to gain deeper insights into the data and the models trained on it. By utilizing these tools, we aim to elucidate the intricate relationships between features and model predictions, providing a transparent and interpretable framework for understanding the factors that drive model decisions.

Permutation feature importance as a technique can be leveraged to quantify the contribution of each feature to the predictive performance of the machine learning models. It can also be visualized using plots. In this study, calculating the importance of the variables provided additional information on how each predictor variable contributed to the performance of the models. This analysis helped identify the key biomarkers and clinical factors that were most predictive of depression severity.

**Fig 7:**
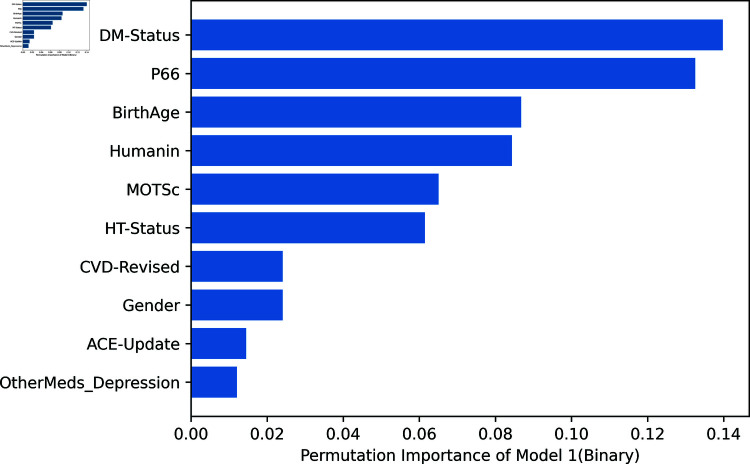
Model 1: All Variables for binary classification.

**Fig 8:**
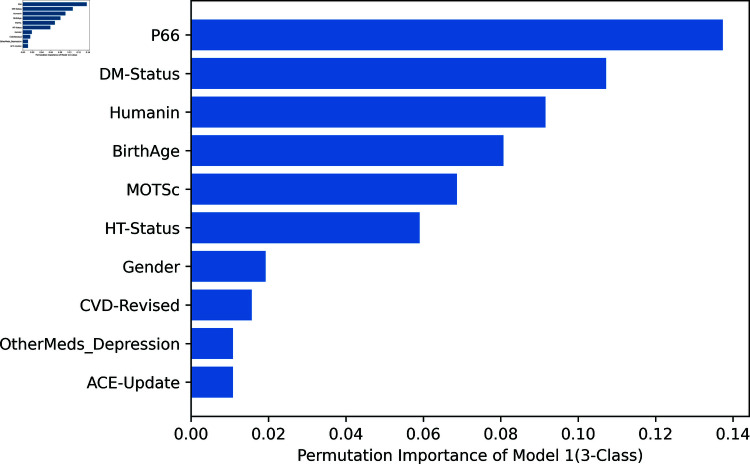
Model 1: All Variables for 3-class classification.

**Fig 9:**
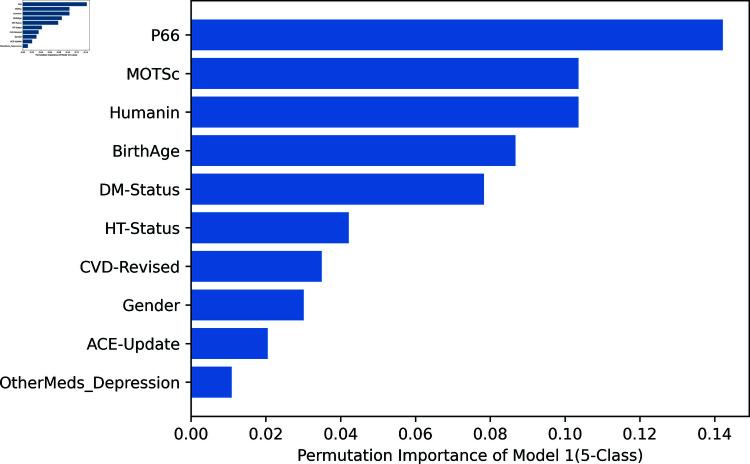
Model 1: All Variables for 5-class classification.

Figs 7–9 depict the permutation importance of features for Model 1 for 2, 3, and 5 classes, respectively using the RF classifier. This analysis shows the similarities and differences in the importance of certain biomarkers (peptides) across these models, as well as in the consistency of the topmost and bottommost variables. The following are noticeable observations: Across all three models, the peptides (P66, Humanin, and MOTSc) consistently rank highly, with P66 often being the top variable. This indicates that these peptides are crucial biomarkers or features across the different classes of classification tasks. The top variable shifts from DM-Status in the 2-class model to P66 in the 3-class and 5-class models, suggesting that as the classification task becomes more complex, P66 becomes increasingly important. Similarly, the least important variable is consistently a feature related to other medications for depression or ACE updates, which may indicate that these factors do not contribute significantly to distinguishing between the classes in the dataset.

**Fig 10:**
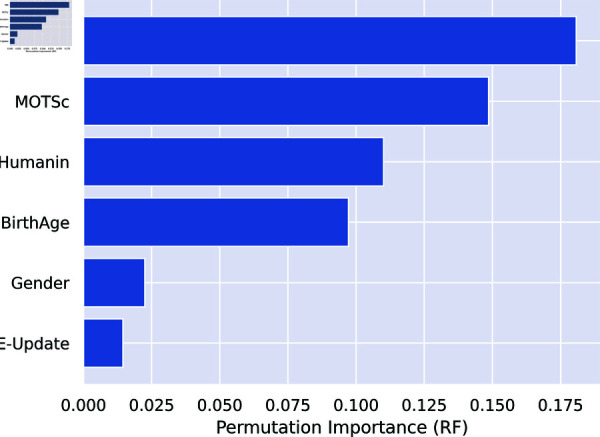
Model 2: Biomarkers + ACE + Age + Gender.

**Fig 11:**
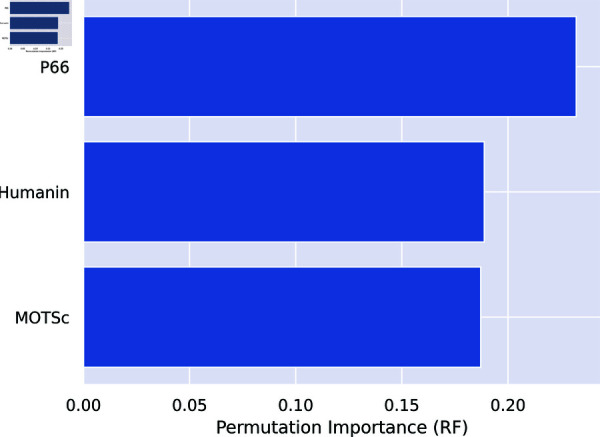
Model 3: Biomarkers only.

Furthermore, Figs 10 and 11 show the permutation importance of features for Model 1 and Model 2 respectively. In Model 2, biomarkers ranked highest on the order of p66shc, MOTS-c, and humanin while ACE ranks least showing that it is the least important variable in predicting the severity of depression in this particular Model. Model 3 indicated that of the three biomarkers of oxidative stress, p66shc has the highest rank, followed by MOTS-c and Humanin.

Beyond the ranking of features or variables using permutation importance, much more can done to understand and interpret data and models. A powerful XAI tool that can be used for this purpose is SHAP [70]. SHAP is a unified framework for interpreting predictions by assigning each feature an importance value (called SHAP values) that reflects its contribution to the model’s output. Derived from cooperative game theory, SHAP values offer a consistent and theoretically sound approach to feature attribution, ensuring that the contributions of all features are fairly represented. In this study, SHAP is utilized to generate both global and local explanations of model 1’s behavior, including feature importance rankings and detailed instance-level breakdowns of prediction contributions.

**Fig 12:**
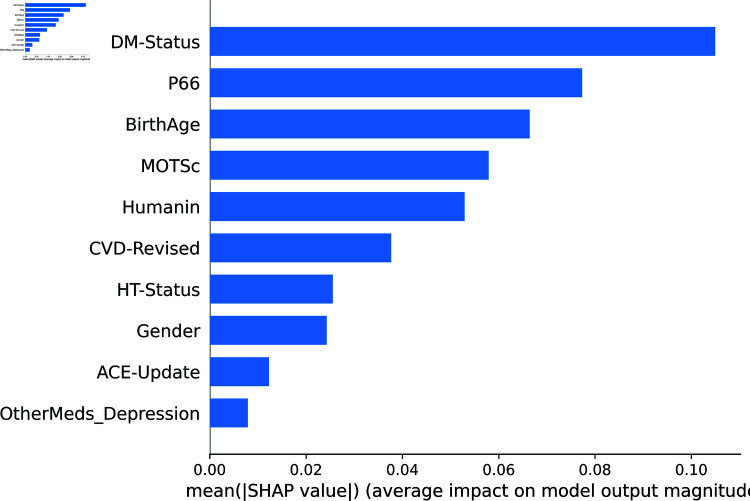
SHAP feature importance for Model 1 (binary classification).

Fig 12 depicts the SHAP summary plot providing an overview of feature importance across all instances in the dataset and ranking them accordingly. Compared with Fig 7 which is the permutation feature importance generated by using the RF classifier, it is very similar. Both plots show that biomarkers (p66shc, MOTS-c, and humanin) are amongst the top-ranked features, while ACE and other medications for depression consistently rank lowest.

**Fig 13:**
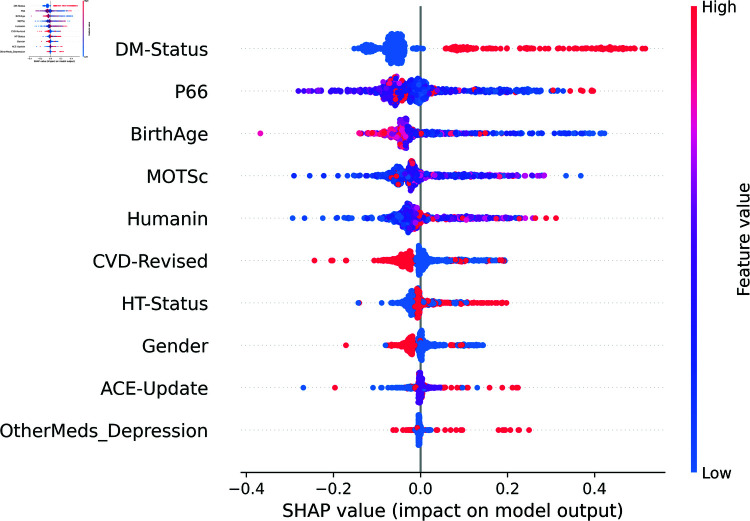
The SHAP Summary plot of Model 1’s Feature effects.

Fig 13 displays the SHAP summary plot that combines feature importance with feature effects across all instances in the training set. The plot sorts features by the sum of SHAP value magnitudes over all samples and uses SHAP values to show the distribution of the impacts each feature has on the model output. The y-axis position is determined by the feature, while the x-axis reflects the corresponding SHAP value. Each point on the plot represents a SHAP value for a specific feature and instance. The plot indicates that “DM-Status,” “P66,” and “BirthAge” are among the most influential features in determining the model’s output, with higher SHAP values indicating a stronger impact on the prediction. The color gradient from blue to red represents the feature values from low to high. For instance, a high “DM-Status” value is associated with a lower SHAP value, indicating a tendency towards predicting Class 0. This plot provides a comprehensive view of how each feature interacts with the model across the dataset, allowing for a deeper understanding of the model’s behavior.

**Fig 14:**
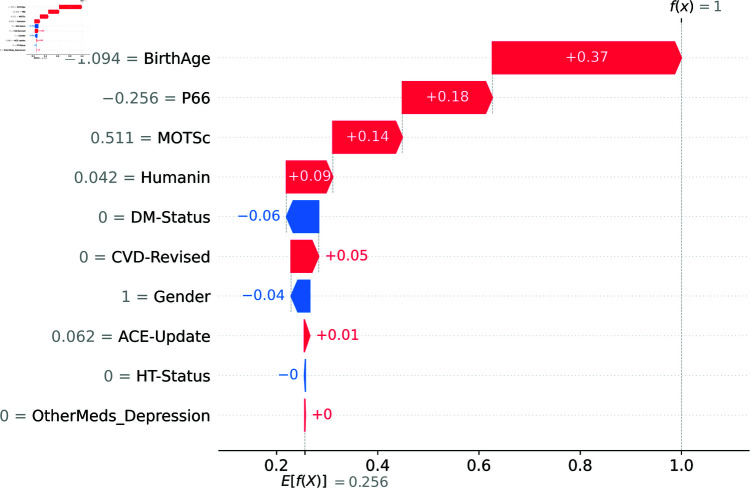
The SHAP Waterfall Plot for Model 1’s Prediction for Instance 6.

Fig 14 is the SHAP waterfall plot showing the breakdown of contributions from individual features to the model’s prediction for Instance 6 (the sixth patient in the training dataset). Each bar represents the impact of a feature, with positive contributions (in red) pushing the prediction towards Class 1 (depressed) and negative contributions (in blue) pulling it towards Class 0 (not depressed) from the base value prediction(E(f(x))=0.256), which is the average prediction the model would make if no features were known or if we considered all instances in the dataset without any specific information about an individual instance. “BirthAge” and “P66” are the most significant contributors, pushing the prediction closer to Class 1, while “DM-Status” and “CVD-Revised” slightly counterbalance this effect by contributing towards Class 0. The final prediction probability (f(x)=1 - indicating that the patient is depressed) is represented at the rightmost point of the plot, summing up the cumulative effects of all features. The values in between (written on either the red or blue bars) indicate the SHAP value of the features. For example, while “BirthAge” contributes to the final prediction by pushing towards it with a SHAP value of 0.37 from the based value prediction, the “DM-Status” pushes away from the final prediction with a SHAP value of 0.06.

Another popular powerful XAI method for explaining individual predictions by approximating the local behavior of complex models with simple, interpretable models, such as linear models is LIME [71]. By perturbing the input data and observing the changes in predictions, LIME generates a set of explanations that highlight which features most significantly contribute to a particular prediction. In this study, LIME is applied to some instances to unravel the reasoning behind specific predictions made by the model.

**Fig 15:**
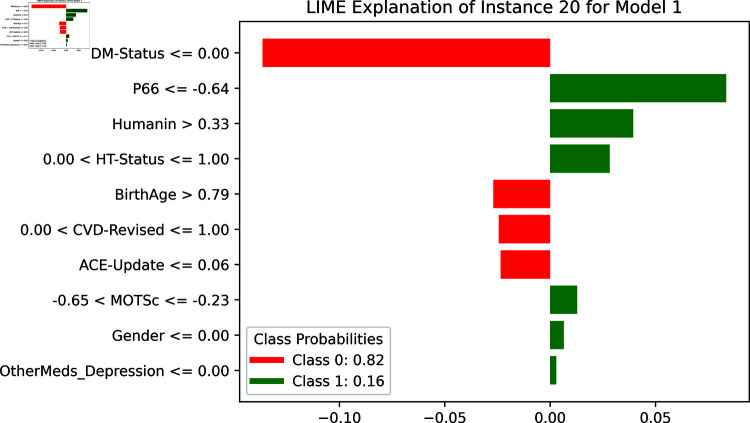
The LIME explanation for Instance 20.

Fig 15 shows the LIME explanation for Instance 20 illustrating the contribution of the top features to the prediction outcome of the binary classification model. The model predominantly associates “DM-Status” with a high likelihood of belonging to Class 0 (not depressed), as indicated by the large negative contribution (red bar). In contrast, features like “P66” and “Humamin” have substantial positive contributions (green bars) toward predicting Class 1 (depressed). This visualization highlights how the model’s prediction for this specific instance is largely driven by the diabetic status (DM-Status), with other features such as P66 and Humamin influencing the probability distribution between Class 0 and Class 1. The class probabilities are presented at the bottom, showing a 0.82 probability for Class 0 and 0.16 for Class 1.

**Fig 16:**
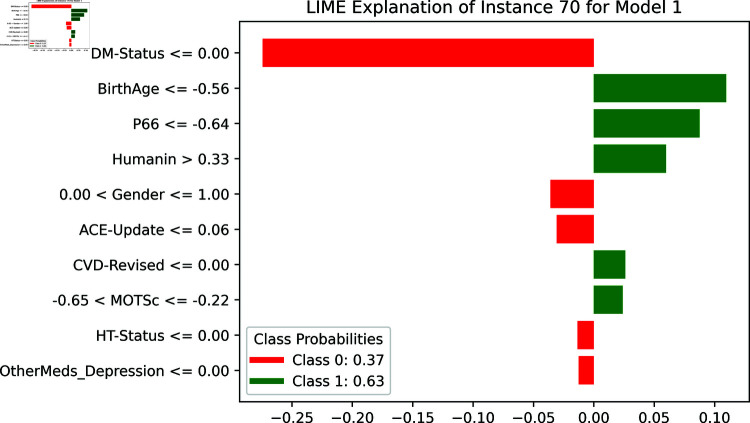
The LIME explanation for Instance 70.

Figure 16 shows the LIME explanation for another instance (Instance 70), detailing the relative contributions of various features to the model’s prediction. Similar to Instance 20, “DM-Status” plays a significant role, but here it is again linked with Class 0. Other features such as “BirthAge” and “P66” show notable negative contributions, pulling the prediction towards Class 0. On the other hand, ”Humamin” and “Gender” contribute positively towards Class 1. The outcome probabilities reveal that this instance has a more balanced prediction, with a probability of 0.37 for Class 0 and 0.63 for Class 1. This suggests that while “DM-Status” heavily influences the prediction, other features also play critical roles, leading to a more nuanced outcome.

### 3.2. Performance evaluation of random forest results on the three models

Tables 6–8 describe our results with a particular focus on the best performing classifier, namely Random Forest (RF). Specifically, Model 3, which exclusively incorporates biomarkers, is compared to Models 1 and 2. Model 1, which covers all variables considered in our experimental setup, consistently exhibits superior precision and F1 scores in all classification categories. This finding underscores the crucial role that a comprehensive set of variables plays in improving predictive accuracy. Model 3, which only includes biomarkers, produces results that are similar to those of Model 1, and even exceeds the performance of Model 1, especially when the data are unbalanced. This similarity in the results between Model 1 and Model 3 demonstrates the influence of oxidative stress biomarkers in the larger model (Model 1) and their association with depression.

**Table 6:** Comparison of the performance evaluation of Random Forest results for binary classification on both balanced and unbalanced data set.

	Unbalanced data set				Balanced data set			
Models	Accuracy	Precision	Recall	F1 Score	Accuracy	Precision	Recall	F1 Score
Model 1	90.96	96.77	68.14	80.00	92.17	91.89	77.27	80.86
Model 2	85.54	85.56	85.54	84.38	86.14	85.78	86.14	83.95
Model 3	89.76	90.12	89.76	89.13	87.35	87.13	87.35	87.2

**Table 7:** Comparison of the performance evaluation of Random Forest results for 3-class classification on both balanced and unbalanced data set.

	Unbalanced data set				Balanced data set			
Models	Accuracy	Precision	Recall	F1 Score	Accuracy	Precision	Recall	F1 Score
Model 1	89.67	89.17	89.76	89.1	90.36	90.5	90.36	90.1
Model 2	88.55	88.8	88.55	87.83	80.12	80.29	80.12	80.12
Model 3	89.16	89.05	89.16	88.56	84.94	86.74	84.94	85.62

**Table 8:** Comparison of the performance evaluation of Random Forest results for 5-class classification on both balanced and unbalanced data set.

	Unbalanced data set				Balanced data set			
Models	Accuracy	Precision	Recall	F1 Score	Accuracy	Precision	Recall	F1 Score
Model 1	87.35	85.64	87.35	85.35	89.76	87.76	89.76	88.26
Model 2	86.14	84.63	86.14	83.63	80.72	79.0	80.72	79.73
Model 3	87.35	87.02	87.35	86.35	79.52	82.76	79.52	80.86

The significance of presenting varying classification results, such as binary, three-class, and five-class classifications, lies in several key aspects that includes:

- Understanding Model Robustness: Evaluating the performance across different classification levels (binary, three-class, and five-class) allows researchers to understand the robustness and generalizability of their models. It helps in determining whether a model that performs well for simpler classifications (e.g., binary) also maintains its performance for more complex classifications (e.g., five-class).
- Benchmarking and Comparisons: The evaluation of the models’ performance across different levels of classification complexity enables benchmarking against other studies or models that might have used different classification schemes. This provides valuable insights into how the models scale as the problem becomes more challenging, transitioning from a simple binary distinction to a more nuanced multi-class categorization.
- Understanding Depression Severity Thresholds and Informing Future Research Directions: This study adhered to the conventional PHQ-9 scoring system, but the supplementary experiments on binary and three-class models indicate that a more refined categorization may be feasible and beneficial. These findings can guide future research by highlighting the need to further investigate the most appropriate level of depression severity classification. It may be worthwhile to explore alternative categorization schemes or feature engineering approaches to improve the performance of the more granular multi-class models.
- Guiding Practical Application: Different real-world applications might require different levels of classification granularity. For instance, a healthcare application might need to distinguish between mild, moderate, and severe depression (three-class) rather than just depression vs. no depression (binary). By showing how models perform across these varying needs, researchers can provide guidance on the most appropriate use cases for their models.

### 3.3. Comparison for balanced and unbalanced data set

The imbalanced data set, comprising binary, 3-class and 5-class classification tasks, obtained accuracy rates of 90. 96%, 89. 67% and 87. 35%, respectively. These promising results established RF as the best classifier algorithm for these classification tasks. When data set balancing techniques were applied, a slight improvement in precision was observed and the F1 score for the RF classifier was recorded as 92. 17%, 90. 36%, and 89. 76% for binary, three-class and five-class classifications, respectively. However, it is important to note that these improvements were observed primarily in Model 1. In contrast, Models 2 and 3 exhibited lower performance for balanced data sets compared to their unbalanced counterparts. One possible explanation for this result is the selective removal of certain variables from Model 1. Consequently, this loss of information can lead to a decrease in overall performance when applied to the balanced data set, as observed in Models 2 and 3. Moreover, it is worth emphasizing that model performance is intricately linked to class balance within the tested data set. In the case of class imbalance, the effectiveness of a model in classifying minority classes may be compromised. This inherent dependence on data balance implies that classification performance is inherently influenced by the balance of the *test* data set.

### 3.4. Implication of the results

The results of this study have several significant implications for the field of mental health, particularly in the prediction and management of depression severity. The implications of these findings extend to clinical practice, preventive healthcare, and ongoing research, ultimately contributing to better management and understanding of depression.

The implications are discussed below:

Clinical implications:

- Biomarker-Based Assessment: The identification of specific mitochondrial-associated oxidative stress biomarkers, such as Humanin, MOTS-c, and p66shc, provides a new avenue for assessing depression severity. Clinicians can use these biomarkers to monitor the progression of depression, enabling more accurate and timely interventions. Also, the high-performing machine learning models, particularly the Random Forest classifier, demonstrate the potential to enhance clinical assessment and diagnosis of depression. By integrating mitochondrial biomarkers and clinical factors, these models can provide more accurate and objective measures of depression severity.
- Treatment Personalization: Understanding the role of these biomarkers allows for personalized treatment plans. Patients exhibiting certain biomarker profiles may respond better to specific treatments, which can lead to more effective management of depression and reduce the trial-and-error approach often associated with prescribing antidepressants. Therefore, the insights into the key predictors of depression severity, including the mitochondrial peptides, can inform the development of more personalized treatment approaches. Targeting specific biological pathways associated with depression may lead to more effective interventions.
- The robust and interpretable machine learning models developed in this study could be translated into practical clinical decision support tools. Such tools could assist healthcare providers in making more informed and personalized assessments and treatment decisions for patients with depression.

Early detection and intervention:

- Predictive Accuracy: With the capability to predict depression severity more accurately, there is potential for early identification of individuals at risk of developing severe depression. Early intervention can prevent the worsening of symptoms and improve long-term outcomes.
- Preventive Strategies: By identifying individuals with elevated levels of specific biomarkers, preventive strategies can be implemented to mitigate the onset or progression of severe depression. This proactive approach can reduce the burden on healthcare systems and improve patient quality of life.

Research implications:

- Expanded Biomarker Exploration: The study highlights the importance of exploring a broader range of biomarkers, including those associated with oxidative stress, inflammation, and neurobiological processes. This can uncover new insights into the underlying mechanisms of depression.
- Mechanistic Understanding: The importance of mitochondrial peptides in predicting depression severity sheds light on the underlying biological mechanisms linking oxidative stress and mitochondrial dysfunction to the development and progression of depressive disorders. This knowledge can guide future research into the pathophysiology of depression.
- Longitudinal Studies: Future research should focus on longitudinal studies with larger and more diverse sample sizes to validate these findings and explore the dynamic nature of depression and its biomarkers over time. This will help in understanding how changes in biomarker levels correlate with disease progression and treatment response.

## 4. Discussion

Mental health research has witnessed a surge in demand for tools that can effectively stratify mental health disorders, such as depression, and provide accurate outcome forecasts. The current study uniquely focused on the impact of mitochondrial-associated oxidative stress biomarkers in this context. In particular, the incorporation of Humanin, MOTS-c, and p66shc within the framework of machine learning techniques has enabled the prediction of the severity of depression based on a binary or multiclass model. Adding information on hypertension, diabetes mellitus, and cardiovascular disease alongside oxidative stress biomarkers added a comprehensive dimension to our predictive models. The combination of these factors reflects a holistic approach to understanding the multifaceted nature of the severity of depression and contributes to the robustness of predictive models. Further, the inclusion of mitochondrial biomarkers has led to a highly accurate assessment of the severity of depression, indicating the importance of mitochondrial oxidative stress in the progression of depressive disorders.

Identification of p66shc, MOTSc, and Humanin as primary biomarkers to predict the severity of depression highlights their biological and clinical relevance [72–75]. Despite the well-established link between elevated oxidative stress and neurological and psychiatric disorders, the inclusion of p66shc, MOTSc, and Humanin as key mitochondrial biomarkers provides new insight into the progression and severity of mental health diseases and strengthens the findings that mitochondrial peptide dynamics play an important role in the pathophysiology of depression, as well as providing an opportunity for a more comprehensive clinical intervention [72].

Machine learning and classification have previously been applied to the classification of depression severity. In a 5-class classification model where the severity of depression was based on the PHQ-9 questionnaire and using social behavior in two different data sets (one from the questionnaire and the other from Twitter social networks), a comparative analysis of different machine learning algorithms showed that the XGBoost classifier gave a precision of 83.87% for the questionnaire-generated data set, while logistic regression resulted in the highest precision of 86.45% for the Twitter tweet data set [76]. Another investigation included length of stay at home, duration of sleep, and vigorous activity among 85 features to predict depression with a Random Forest and a set of SVM and KNN and resulted in an equivalent precision of 90% [77]. Similarly, a study using the Depression, Anxiety, and Stress Scale Questionnaire (DASS-21) on 348 participants and five depression categories applied Naive Bayes and achieved a precision of 85.5% for the detection of depression [78]. Furthermore, a comprehensive investigation was carried out that involved six different machine learning classifiers using various sociodemographic and psychosocial information from 604 participants to detect depression. AdaBoost classifier with the SelectKBest feature selection technique outperformed all other classifiers with an accuracy of 92.56% [79]. These collective findings, along with the current study, underscore the growing consensus on the efficacy of machine learning in discerning intricate patterns and predicting depression outcomes.

It should be noted that this study represents a novel approach to the use of specific mitochondrial-associated oxidative stress biomarkers within the framework of machine learning to predict the severity of depression. The inclusion of Humanin, MOTS-c, and p66Shc in the analysis represents a novel avenue that extends the application of machine learning techniques in mental health research.

Although ACE has previously been associated with DM, its inclusion in our study aims to explore its potential relevance to the severity of depression. The rationale lies in the intricate interplay between the renin-angiotensin system (RAS), of which ACE is a key component, and various physiological processes, including those involved in mental health. Emerging evidence suggests that RAS, beyond its traditional role in cardiovascular regulation, may have a modulatory effect on neurotransmission and neuroendocrine pathways [3,80]. Dysregulation of RAS has also been implicated in neuroinflammation, oxidative stress, and altered cerebral blood flow, all factors associated with depression. Furthermore, ACE inhibitors, commonly used to regulate blood pressure by targeting RAS, have shown neuroprotective effects and potential benefits in neurodegenerative disorders [3,80]. Although the specific link between ACE and depression is still an area of active investigation, the potential influence of ACE on neurobiological processes makes it a relevant candidate for exploration in the context of predicting the severity of depression [81,82]. However, in the current study, ACE genotype appears to be of relatively low importance in predicting the severity of depression. Several reasons can explain this observation. The ACE genotype may have a stronger association with hypertension, diabetes, and cardiovascular disease, which is not reflected when these diseases are included as comorbidities in the models [83,84]. Mitochondrial peptides and clinical factors played a more significant role in predicting disease severity compared to the ACE genotype in the current study. This suggests that the ACE genotype may have a limited impact on disease progression compared to other factors. Furthermore, the ACE genotype may interact with other genetic or environmental factors to influence the severity of the disease. By including ACE in this study, the objective was to contribute to an evolving understanding of the multifaceted connections between cardiovascular health, neurobiology, and mental health outcomes. More research is needed to elucidate the specific mechanisms through which ACE can impact depression severity and establish its potential as a biomarker to predict mental health disorders.

The difference between this study and previous research can be attributed to the use of new biomarkers, the specific focus of the study, and the limitations of previous research. Additionally, variability in study designs, sample populations, and methodologies in different investigations introduces a layer of complexity when attempting to compare results. Participant diversity, diagnostic criteria, and data analysis techniques can contribute to discrepancies between studies. Furthermore, the retrospective nature of some studies, including potential biases and confounding factors, may impact the generalizability of the findings.

Future studies with larger and more diverse sample sizes, as well as longitudinal designs, will provide a more comprehensive understanding of the intricate relationships between biomarkers and the severity of depression. The current study was limited by its relatively small sample size and cross-sectional nature. Larger studies with more participants from diverse backgrounds would help validate the findings and determine if the associations hold true across different populations. Longitudinal studies that follow participants over time would shed light on how changes in biomarker levels relate to changes in depression severity. This would help clarify the directionality of the relationships and whether biomarkers can predict future depression risk or track responses to treatment.

An expanded focus on biological markers associated with oxidative stress, inflammation, and neurobiological processes, including peripheral biomarkers such as cytokines, growth factors, hormones, and protein markers, could further enhance understanding of the underlying pathophysiology of depression. Incorporating behavioral data from smartphones and wearable technologies, including activity levels, sleep patterns, dietary habits, and social interactions, also holds promise for capturing the real-time dynamics of depressive symptoms and their interaction with biological processes. Furthermore, delving into genetic, molecular and neuroimaging factors, as well as exploring the interactions between the hypothalamic-pituitary-adrenal (HPA) axis and mitochondrial metabolism, offers the potential to uncover novel predictors and improve the overall robustness of predictive models for the severity of depression. While this study provides promising preliminary evidence, further research is needed to fully elucidate the complex interplay between mitochondrial peptides, clinical factors, and depression.

## 5. Conclusion

This study highlights the potential of machine learning (ML) techniques in conjunction with biomarkers to diagnose and understand the progression of depression. The findings highlight the considerable impact of Humanin, MOTS-c, and p66shc on predicting the severity of depression. In the context of clinical applications, the study has implications for practitioners who want to use ML and biomarkers to assess and manage the severity of depression.

## Supporting information

S1 Text Supplementary material. This file contains Tables S1 to S9.S1 to S3 are Tables 9–11 showing the results for all classes for Model 1.S4 to S6 are Tables 12–14 showing the results for all classes for Model 2.S7 to S9 are Tables 15–17 showing the results for all classes for Model 3.
(TEX)

## Footnotes

1 ROS is a group of highly reactive molecules that contain oxygen, such as superoxide, hydrogen peroxide, and hydroxyl radicals. These molecules can cause damage to cellular components, including DNA, proteins, and lipids.
